# The Detection of Retina Microvascular Density in Subclinical Aquaporin-4 Antibody Seropositive Neuromyelitis Optica Spectrum Disorders

**DOI:** 10.3389/fneur.2020.00035

**Published:** 2020-02-11

**Authors:** Yihong Chen, Ce Shi, Lili Zhou, Shenghai Huang, Meixiao Shen, Zhiyong He

**Affiliations:** ^1^Department of Neurology, The Second Affiliated Hospital and Yuying Children's Hospital of Wenzhou Medical University, Wenzhou, China; ^2^School of Ophthalmology and Optometry, Wenzhou Medical University, Wenzhou, China

**Keywords:** neuromyelitis optica spectrum disorders, optical coherence tomography angiography, diagnosis, aquaporin-4 antibody, optic neuritis

## Abstract

**Purpose:** To use optical coherence tomography (OCT) and OCT angiography (OCT-A) to measure changes in the retinal structure and microvasculature of patients with aquaporin-4 antibody-positive, neuromyelitis optica spectrum disorder (NMOSD) with a history of optic neuritis (NMOSD+ON) and those without it (NMOSD–ON).

**Methods:** A total of 27 aquaporin-4 antibody-positive NMOSD patients and 31 age- and gender-matched healthy control (HC) participants were included. In 27 NMOSD patients, 19 of them had a history of optic neuritis (ON) and 8 of them had no history of ON. Peripapillary retinal nerve fiber layer (pRNFL) thickness and macular ganglion cell and inner plexiform layer (GCIPL) thickness were measured by OCT. Radial peripapillary capillary density (RPCD) and macular superficial vessel density (MSVD) were measured by OCT-A. Comparisons of retinal structural and microvascular parameters between the cohorts were performed using generalized estimating equation (GEE) models. Diagnostic accuracy was evaluated by the area under the receiver operating characteristics curve (AROC).

**Results:** In NMOSD+ON eyes, the GCIPL and pRNFL thicknesses, 48.6 ± 7.1 and 61.7 ± 25.1 μm, respectively, were significantly thinner than in HC eyes (*P* < 0.001 for both). However, in NMOSD–ON eyes, the GCIPL and pRNFL thicknesses were not significantly thinner than in HC eyes (*P* > 0.05 for both). In NMOSD+ON eyes, the RPCD and MSVD, 37.8 ± 7.1 and 36.7 ± 5.0%, respectively, were significantly less dense than HC eyes (*P* < 0.001 for both). Similarly, the RPCD and MSVD in NMOSD–ON eyes, 49.0 ± 2.8 and 43.9 ± 4.2%, respectively, were also less dense than in HC eyes (*P* < 0.029 for RPCD, *P* < 0.023 for MSVD). The highest AROC, 0.845 (sensitivity = 88.5%, specificity = 78.0%), was achieved by the logistic regression combination of all of the variables, i.e., pRNFL, GCIPL, RPCD, and MSVD.

**Conclusions:** Retinal microvascular changes were present in NMOSD–ON eyes. The combination of retinal structural and microvascular parameters might be helpful to discriminate NMOSD–ON eyes from HC eyes.

## Introduction

Neuromyelitis optica spectrum disorders (NMOSDs) are autoimmune inflammatory conditions of the central nervous system (CNS) with a frequently relapsing course that predominantly affects the optic nerves and spinal cord (1). The aquaporin-4 antibody (AQP4-ab), discovered in 2004 (2), can be detected in 60–80% of NMOSD patients and has enabled the rapid evolution of our understanding of the immunopathogenesis of this disease (3, 4).

Approximately 80% of NMOSD patients have severe visual impairment and even blindness after an acute episode of optic neuritis (ON) (5–7). However, the time of the first onset of ON in NMOSD varies among patients, and until the first episode, few morphological changes are evident in fundus photographs. Prior to the onset of clinical NMOSD symptoms, subclinical disease activity is not generally evident. However, a recent study using optical coherence tomography (OCT) reported foveal thinning irrespective of ON in NMOSD patients (8). This indicates the presence of subclinical primary retinal pathology. The presumed primary retinal pathology could benefit quicker diagnosis and tracking of disease development in NMOSD; however, research in this regard is still limited (9).

OCT can be used to measure the thickness of the peripapillary retinal nerve fiber layer (pRNFL) and the ganglion cell and inner plexiform layer (GCIPL). Studies utilizing OCT have revealed profound thinning of the pRNFL and GCIPL in NMOSD-related ON eyes, indicating retinal axonal and neuronal loss (10–14). Recently, a study reported that vascular changes, including attenuation of the peripapillary vascular tree and focal arteriolar narrowing, can be seen on fundoscopy after NMOSD ON (15), suggesting that retinal vascular damage may also play a role in the NMOSD process. In other similar diseases, such as multiple sclerosis, composite indicators of blood flow and structure can better detect retinal damage (16). Hence, OCT-based exploration of the structural and vascular measurements might provide image biomarkers that indicate the presence of NMOSD in eyes, even in ones without ON.

OCT-angiography (OCT-A) can also detect changes in the retinal microvasculature. In this study, we used OCT and OCT-A to detect retinal structural and microvascular damage in AQP4-ab-positive NMOSD eyes with or without a history of ON.

## Materials and Methods

### Study Population

Ethical approval for this study was obtained from the Institutional Ethics Committee at Wenzhou Medical University, and written informed consent was obtained from each participant in accordance with the Declaration of Helsinki. In this cross-sectional study, 27 patients with NMOSD were enrolled from The Second Affiliated Hospital, Wenzhou Medical University, Wenzhou, China. In our study population, all of the NMOSD patients were females, which is consistent with the known prevalence of this disease among males and females (17, 18). Thirty-one age- and gender-matched healthy participants, who were all working staff from The Affiliated Eye Hospital of Wenzhou Medical University, were enrolled as the healthy control (HC) group.

### Diagnostic Criteria

The patients enrolled were diagnosed with NMOSD by a single neurologist (Zhiyong He, MD) based on the 2015 diagnostic criteria of the International Panel for Neuromyelitis Optica Diagnosis (19). ON was defined according to the guidelines of the Optic Neuritis Treatment Trial (20). The number of ON episodes was obtained from each patient's medical record. All patients underwent orbital MRI with T2-weighted image and gadolinium-enhanced T1 sequences. All patient sera were analyzed for AQP4-ab by indirect immunofluorescence at a branch of the Euroimmun Medical Diagnostic Laboratory in China (EUROIMMUN AG, Lübeck, Germany).

The inclusion criteria of patients were as follows: best corrected visual acuity (BCVA) ≥ 20/400; intraocular pressure (IOP) ≤ 21.0 mmHg; refraction error between +3.00 diopters (D, hyperopia) to−5.00 D (high myopia); no ON attack within the last 6 months before enrollment; able to perceive the light spot during OCT and OCT-A examinations and cooperate with the examiner; no history of ocular surgery or other eye diseases (glaucoma, age-related macular degeneration, etc.); and serum AQP4-ab positive. Patients with AQP4-ab positive but no ON had isolated or recurrent myelitis. The inclusion criteria of HCs were similar to those for the NMOSD group except for the BCVA ≥ 20/25, presence of ON, and the absence of serum AQP4-ab.

Clinical characteristics including disease history (hypertension, dyslipidemia, heart disease, and diabetes), history of smoking, history of ON, number of ON episodes, and Expanded Disability Status Scale (EDSS) scores (21) were obtained. All study participants underwent a complete ophthalmologic examination including assessment of BCVA, refraction, IOP, pupillary reflexes, OCT, OCT-A, and a dilated slit-lamp biomicroscopy with fundus examination. Reproducibility of the OCT and OCT-A data acquisition was validated well as previously reported (22). In addition, our research team has published a few papers using this method and also showed good reproducibility (23, 24). Furthermore, one trained and experienced technician performed the OCT and OCT-A measurements to ensure reproducibility of the data acquisition. Blood pressure was well-controlled in all participants with hypertension.

### OCT Structure

**Figure 1:**
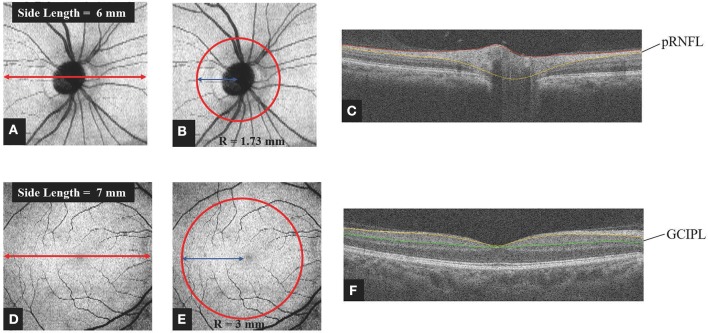
Method of obtaining retinal thickness. **(A)** Representative image captured by the 3D Disc protocol of spectral domain OCT. The image was centered on the optic disc and covered a square grid (6 × 6 mm). **(B,C)** For analysis of the pRNFL thickness, further segmentation was done automatically by layering software that was centered on the optic disc and covered a circle 1.73 mm in radius. Manual correction was not employed. **(D)** Representative image captured by the 3D Retina protocol of spectral domain OCT. The image was centered on the macula and covered a square grid (7 × 7 mm). **(E,F)** For analysis of the GCIPL thickness, further segmentation was done automatically by the layering software. The image was centered on the macula and covered a circle 3 mm in radius. Manual correction was not employed. OCT, optic coherence tomography; pRNFL, peripapillary retina nerve fiber layer; GCIPL, ganglion cell layer and inner plexiform layer; R, radius.

pRNFL and GCIPL thickness measurements were performed with an RTVue-XR Avanti spectral-domain OCT (Optovue, Inc., Fremont, CA, USA; software version 2017.1.0.155). Details of this device have been published (25, 26). For analysis of the pRNFL thickness, we obtained images by the three-dimensional (3D) disc protocol, which was centered on the optic disc and covered a square grid of 6 × 6 mm (Figure 1A). Further segmentation of the optic disc covered a 1.73-mm-radius circle (Figure 1B). It was performed automatically by the retinal segment software (OCTExplorer, University of Iowa, IA, USA, Software V.3.8.0) (27–30) and required no manual correction (Figures 1B,C). This segmentation software is a separate one from built-in software of OCT-A. In this segmentation software, 10 intraretinal layers are first automatically segmented and the 3-D image dataset is flattened to remove motion-based artifacts. After running this software, we can obtain the thickness of each layer of retina. The GCIPL thickness was acquired using the 3D retina scanning protocol of the built-in software. The measurements were centered on the macula and covered a square grid of 7 × 7 mm (Figure 1D). Further segmentation was done automatically by the same retinal segment software as described for the pRNFL. The segmentation was centered on the macula and covered a 3-mm-radius circle and required no manual correction (Figures 1E,F). Only images with a scan quality of at least 6 were included for further analysis.

### OCT-A

**Figure 2:**
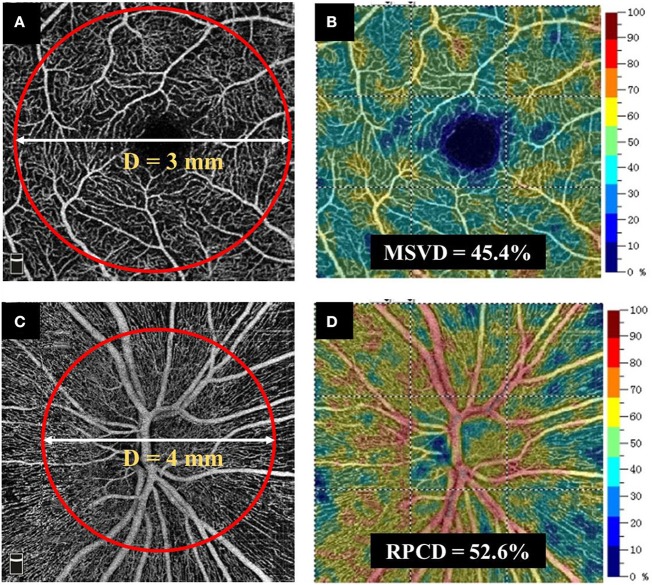
Method for obtaining retinal vascular density. **(A,B)** MSVD was measured using the RTVue-XR Avanti spectral-domain OCT. MSVD was acquired through scans within the annular zone of 3 mm diameter around the center of the macula, and the superficial capillary layer from 3 μm below the ILM to the outer boundary of the inner plexiform layer was analyzed. **(C,D)** An en face angiogram of the radial peripapillary capillary segment was obtained by the maximum flow (decorrelation value) projection that extended from the ILM to the posterior boundary of RNFL. It covered a 4.5 × 4.5 mm field of view centered on optic disc. The RPCD data were automatically measured by the built-in software in a 4-mm-diameter circle centered on the optic disc. MSVD, macular superficial vessel density; RPCD, radial peripapillary capillary density; OCT, optic coherence tomography. ILM, internal limiting membrane; OCT-A, optic coherence tomography angiography.

Radial peripapillary capillary density (RPCD) and macular superficial vessel density (MSVD) were measured using the RTVue-XR Avanti spectral-domain OCT. In brief, the total scan time was <3 s to minimize the motion artifacts. A 3D OCT scan pattern was obtained, and the split-spectrum amplitude decorrelation angiography algorithm was used to extract signals of blood cell motion (31). After a series of processing methods, an en face algorithm was performed to refine the retinal capillary images from the macula and optic nerve head for density analysis. In the 4.5 × 4.5 mm field of view centered on the optic disc, the radial peripapillary capillary segments extended from the internal limiting membrane (ILM) to the posterior boundary of retinal nerve fiber layer (RNFL). The MSVD was acquired through scans within the 3-mm-diameter annular zone around the macular center (Figure 2A). The superficial capillary layer, located from 3 μm below the ILM to the outer boundary of the inner plexiform layer, was analyzed (Figures 2A,B). The RPCD data were automatically measured by the built-in software in a 4-mm diameter circle centered on the optic disc (Figures 2C,D). The vessel density was also obtained from the OCT-A built-in software. Images with a scan quality <6 or with residual motion artifacts were excluded from the image analysis.

### Statistical Analysis

All data analyses were performed by SPSS (Statistical Package for the Social Sciences, Ver. 22, IBM, Armonk, NY, USA) for Windows and MedCalc V.10.1.3.0 f (MedCalc Software, Ostend, Belgium, www.medcalc.be). For continuous variables, the means ± standard deviations were calculated. Three groups were analyzed: (1) eyes with a history of ON (NMOSD+ON), (2) eyes without a history of ON (NMOSD–ON), and (3) HC eyes. Differences in basic ophthalmic parameters among the cohorts were tested using one-way analysis of variance or Kruskal-Wallis test. Comparisons of spectral domain OCT parameters and OCT-A parameters between the cohorts were performed using the generalized estimating equation (GEE) models to account for intrasubject inter-eye dependencies. Correction for multiple comparisons was automatically performed by the GEE models. We also compared all variables we measured between unaffected eyes of NMOSD patients with a history of ON (NMOSD unilateral-ON group) and eyes of NMOSD patients without a history of ON (NMOSD non-ON group) using GEE models. Except for the axial length (NMOSD unilateral-ON group = 23.6 mm, NMOSD non-ON group = 22.8 mm, *P* = 0.01), other variables were not statistically different between two groups. The area under the receiver operating characteristic curve (AROC) was used to calculate the power of each diagnostic parameter. Logistic regression was employed to combine diagnostic parameters into diagnostic indices in the GEE models. Statistical significance was set at *P* < 0.05.

## Results

### Demographics

A total of 27 patients with NMOSD (54 eyes) and 31 age-matched HC participants (62 eyes) were enrolled. In 27 NMOSD patients, 19 of them had a history of ON, and 8 of them had no history of ON. Seven NMOSD eyes were excluded due to low signal strength. Among them, five eyes were excluded due to poor visual acuity and two eyes were excluded due to poor fixation. Therefore, we compared 47 NMOSD eyes (21 ON and 26 without ON) with 62 HC eyes. The clinical characteristics and basic ophthalmic information of the participants are shown in Tables 1, 2, respectively.

**Table 1:** Participant demographics and clinical summary.

	**NMOSD (*n* = 27)**	**HC (*n* = 31)**
Gender (Male/Female)	0/27	0/31
Age, years (years)	50.2 ± 12.5	47.7 ± 13.2
BMI	23.0 ± 3.1	22.9 ± 2.6
Median EDSS (range)	3.0 (0.0–7.0)	–
Medical history, *n* (%)		
Hypertension	3 (10.7)	1 (3.2)
Dyslipidemia	1 (3.6)	6 (19.4)
Heart disease	0 (0.0)	0 (0.0)
Diabetes	0 (0.0)	0 (0.0)
Smoking	1 (3.6)	1 (3.2)
Bilateral ON, *n* (%)	7 (25.9)	–
Unilateral ON, *n* (%)	12 (44.4)	–
No history of ON, *n* (%)	8 (29.6)	–

*NMOSD, neuromyelitis optica spectrum disorders; HC, healthy controls; BMI, body mass index; EDSS, Expanded Disability Status Scale; n, number of participants; ON, optic neuritis*.

**Table 2:** Basic ophthalmic assessments of NMOSD–ON, NMOSD+ON, and HCs.

	**NMOSD–ON**	**NMOSD+ON**	**HC**	***P*_**among three groups**_**
Number of eyes	26	21	62	–
AL, mm	23.11 ± 0.63	23.69 ± 0.75	23.60 ± 1.25	0.289^a^
BCVA, LogMAR	0.00 ± 0.11	0.31 ± 0.43	−0.04 ± 0.07	<0.001^b^
BCVA, Snellen decimal	1.03 ± 0.12	0.68 ± 0.38	1.12 ± 0.18	<0.001^b^
SE, D	−0.22 ± 1.65	−0.28 ± 1.07	−0.52 ± 1.72	0.814^a^
IOP, mmHg	13.3 ± 3.0	12.7 ± 1.7	12.3 ± 1.9	0.189^b^
Frequency of ON, *n* (%)				
None	26 (55.3)	–	–	–
1 time	–	9 (19.1)	–	–
≥2 times	–	12 (25.5)	–	–

*NMOSD–ON, neuromyelitis optica spectrum disorders patients without a history of optic neuritis; NMOSD+ON, neuromyelitis optica spectrum disorders patients with a history of optic neuritis; HC, healthy controls; AL, axial length; BCVA, best corrected visual acuity; SE, spherical equivalent; D, diopter; IOP, intraocular pressure; ON, optic neuritis*.

a *ANOVA*.

b *Kruskal-Wallis test*.

### Comparison of OCT Structural Parameters

#### pRNFL Thickness

**Figure 3:**
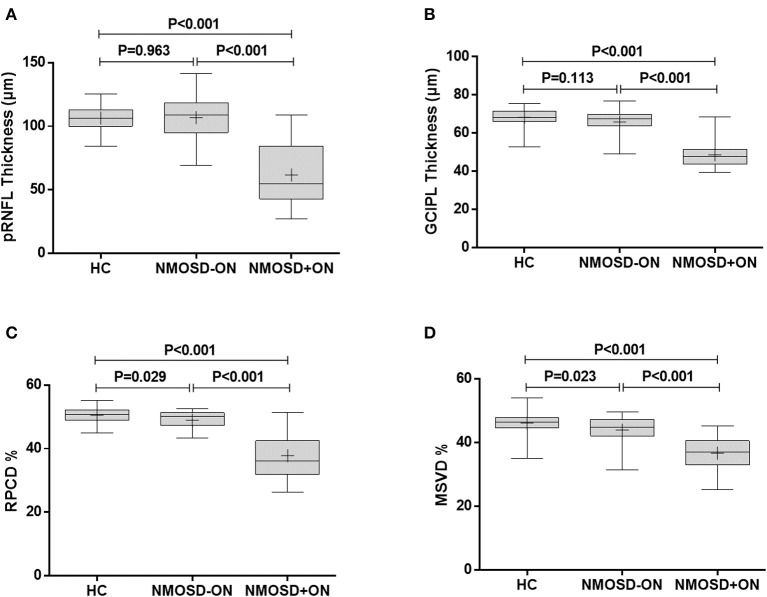
Comparison of retinal thickness and vascular density between NMOSD and HC. **(A)** The comparison of pRNFL thickness, **(B)** GCIPL thickness, **(C)** RPCD, and **(D)** MSVD among the healthy control eyes, NMOSD eyes without a history of ON, and NMOSD eyes with a history of ON. HC, healthy control; NMOSD–ON, neuromyelitis optica spectrum disorders without optic neuropathy; NMOSD+ON, neuromyelitis optica spectrum disorders with optic neuropathy; pRNFL, peripapillary retina nerve fiber layer; GCIPL, ganglion cell layer and inner plexiform layer; RPCD, radial peripapillary capillary density; MSVD, macular superficial vessel density. Box, the upper quartile to the lower quartile values; +, mean value; bar inside box, median value; vertical lines, maximum and minimum values.

The pRNFL thickness in the NMOSD+ON eyes, 61.7 ± 25.1 μm, was significantly thinner than in the NMOSD–ON and HC eyes (106.9 ± 16.5 and 106.4 ± 9.3 μm, respectively; *P* < 0.001; Figure 3A). However, the pRNFL thickness in the NMOSD–ON eyes was not different from the HC eyes (*P* = 0.963).

#### GCIPL Thickness

Similarly, the GCIPL thickness in the NMOSD+ON eyes, 48.6 ± 7.1 μm, was thinner than in the NMOSD–ON and HC eyes (65.9 ± 6.3 and 68.3 ± 4.4 μm, respectively; *P* < 0.001 for both, Figure 3B). The GCIPL thickness of the NMOSD–ON group was not significantly different from the HC group (*P* = 0.113).

### Comparison of OCT Microvascular Parameters

#### RPCD

The RPCD in the NMOSD+ON eyes, 37.8 ± 7.1%, was lower than that in the NMOSD–ON and HC eyes (49.0 ± 2.8 and 50.5 ± 2.2%, *P* < 0.001 for both, Figure 3C). Moreover, the RPCD was lower in the NMOSD–ON eyes than in the HC eyes (*P* = 0.029).

#### MSVD

The MSVD in the NMOSD+ON eyes, 36.7 ± 5.0%, was significantly lower than in the NMOSD–ON and HC eyes, 43.9 ± 4.2 and 46.1 ± 3.4 %, respectively (*P* < 0.001 for both; Figure 3D). Furthermore, the MSVD was also lower in the NMOSD–ON eyes than in the HC eyes (*P* = 0.023).

#### AROC Curve Analysis

**Figure 4:**
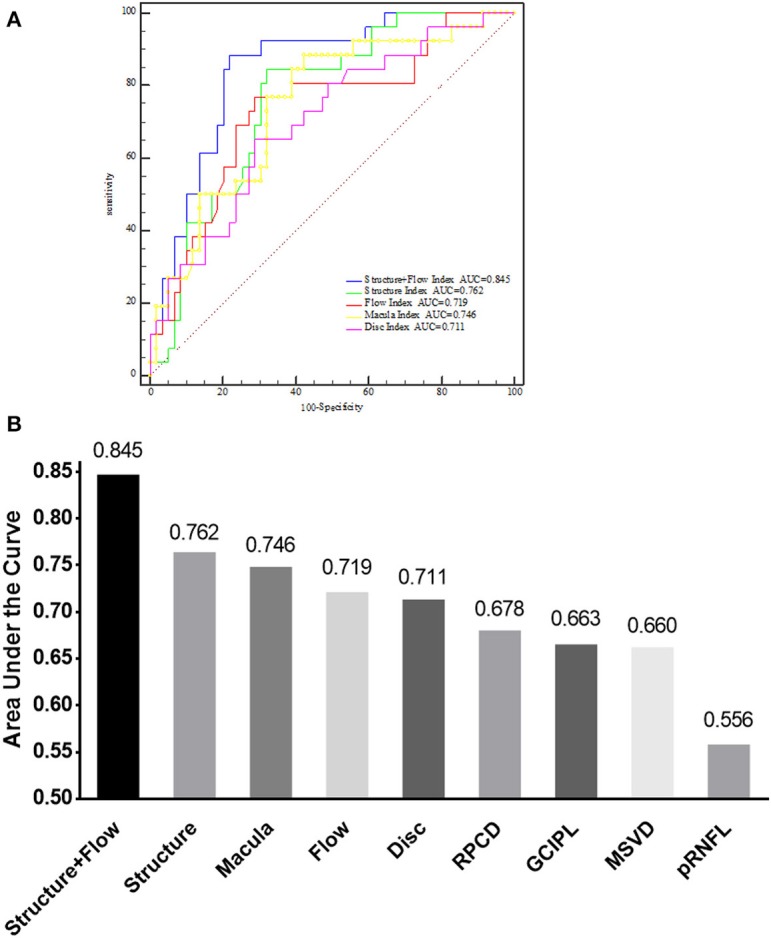
AUC of different indices for discriminating between NMOSD–ON and HC. **(A)** Comparison of the Structure+Flow index, Structure index, Flow index, Macula index, and Disc index by receiver operating characteristic curves analysis for discriminating between NMOSD–ON and HC eyes. **(B)** AUC of nine different parameters for discriminating between NMOSD–ON and HC eyes. NMOSD–ON, neuromyelitis optica spectrum disorders without a history of optic neuritis. NMOSD+ON, neuromyelitis optica spectrum disorders with a history of optic neuritis; HC, healthy control; AUC, area under the curve; GCIPL, ganglion cell layer and inner plexiform layer; pRNFL, peripapillary retina nerve fiber layer; MSVD, macular superficial vessel density; RPCD, radial peripapillary capillary density. Structure, a combination of GCIPL and pRNFL; Flow, a combination of MSVD and RPCD; Structure+Flow, a combination of MSVD, RPCD, pRNFL, and GCIPL; Disc, a combination of RPCD and pRNFL; Macular, a combination of MSVD and GCIPL.

Analysis of the AROC curve was used to determine the ability of OCT and OCT-A parameters to discriminate the NMOSD–ON eyes from HC eyes. The AROC-determined sensitivity and specificity of these parameters were based on the construction by logistic regression of the Structure+Flow index, a combination of the OCT structure index (which itself was a combination of pRNFL and GCIPL thickness) and the OCT-A flow index (which itself was a combination of MSVD and RPCD). The Structure+Flow index area under the curve (AUC) was 0.845 with a sensitivity of 88.5% and a specificity of 78.0% (*P* < 0.001, Table 3, Figures 4A,B).

**Table 3:** Diagnostic accuracy of OCT and OCT-A parameters.

**NMOSD–ON vs. HC**						
	**AUC**	**95% CI**	**AUC *p***	**Cutoff Point**	**Sensitivity, %**	**Specificity, %**
pRNFL	0.556	0.420–0.693	0.418	≤2.339	23.08	93.55
MSVD	0.660	0.543–0.784	0.008	≤2.51	88.46	44.07
GCIPL	0.663	0.533–0.787	0.013	≤2.421	69.23	61.29
RPCD	0.678	0.559–0.797	0.003	≤2.472	80.77	51.61
Disc = pRNFL+RPCD	0.711	0.593–0.830	<0.001	≤2.29	65.38	72.58
Flow = MSVD+RPCD	0.719	0.599–0.839	<0.001	≤2.402	76.92	69.35
Macula = GCIPL+MSVD	0.746	0.633–0.859	<0.001	≤2.478	88.46	57.63
Structure = GCIPL+pRNFL	0.762	0.659–0.866	<0.001	≤2.413	84.62	67.80
Structure+Flow	0.845	0.759–0.931	<0.001	≤2.383	88.46	77.97

*Logistic regression was employed to combine diagnostic parameters into composite diagnostic indices. OCT, optical coherence tomography; OCT-A, optical coherence tomography angiography; NMOSD, neuromyelitis optica spectrum disorders; ON, optic neuritis; AUC, area under the curve; CI, confidence interval; AUC p, p value of area under the curve; GCIPL, ganglion cell layer and inner plexiform layer; pRNFL, peripapillary retina nerve fiber layer; MSVD, macular superficial vessel density; RPCD, radial peripapillary capillary density*.

## Discussion

In this study, we characterized the retinal structural and microvascular changes in NMOSD eyes with and without a history of ON. The retinal pRNFL and GCIPL thicknesses in NMOSD–ON eyes and HC eyes were not different from one another; however, both layers were thinner in the NMOSD+ON eyes compared to HC eyes. The microvascular densities in both the macula and the optic nerve head were lower in NMOSD eyes with or without ON compared to HC eyes. Studying the structural and microvascular changes can help to understand the neurodegenerative process in NMOSD patients. Our results showed that the combined structural and microvascular indices had good discriminative power to differentiate NMOSD–ON from HC eyes.

Both the pRNFL and the GCIPL thicknesses of the NMOSD+ON group were remarkably reduced compared to the HCs, and this is consistent with previous studies (8, 32). There were no significant differences in the pRNFL and GCIPL thicknesses between the NMOSD–ON group and HCs. However, a larger, previously published study reported the presence of abnormalities in GCIPL thickness in NMOSD–ON eyes (33). Thus, it is possible that our current study did not detect significant differences in the OCT measurements between NMOSD–ON and HC eyes due to the small size of the study population. The decreased thickness of pRNFL and GCIPL indicated that neuro-axonal and ganglion cell damage existed in NMOSD+ON patients.

Recurrent ON in NMOSD causes severe thinning of pRNFL and GCIPL thickness (34), and this has been considered to be a reliable imaging biomarker for NMOSD (35). Based on our results, we speculated that the thinning of pRNFL and GCIPL might be related to abnormalities of the retinal microvascular circulation. A pathology study of NMOSD patients found that the vascular walls of the optic nerve were infiltrated by inflammatory cells (36). It is likely that inflammation-induced damage to astrocytes and vascular endothelial cells leads to a direct reduction of vascular perfusion that could facilitate neurodegeneration. However, the relationship between retinal microvascular perfusion and NMOSD-related structural changes in the retina needs further long-term studies.

In contrast to our pRNFL and GCIPL thickness results, the retinal vessel density as measured by the MSVD and RPCD was remarkably reduced in NMOSD eyes with a history of ON and mildly reduced in NMOSD eyes without a history of ON, as recently described (33). This suggests that subclinical primary retinal vasculopathy exists in NMOSD, and retinal vascular changes may play an important role in this disorder. In addition, a recent study provided structural and functional evidence of Müller glial dysfunction in eyes of patients with AQP4-ab-positive NMOSD (37). The reason for ON-independent vessel rarefaction in NMOSD is unclear. One possible explanation is that Müller cells might be the target of direct attack by AQP4-ab in NMOSD before the occurrence of ON. NMOSD is regarded as a primary astrocytopathy (35). Müller cells are responsible for water homeostasis, energy metabolism, neurotransmitter recycling, and maintenance of the blood-brain barrier function. These retinal astrocytic cells, especially those in the inner nuclear layer, express high levels of aquaporin-4 (38, 39). Moreover, the phenomenon of astrocyte-associated vascular change has been reported in other research models (40–42). Thus, it is possible that Müller cells are likely to be attacked by AQP4-ab before the development of ON. Such attacks could affect the function of energy metabolism, resulting in the retinal vascular rarefaction in NMOSD.

Interestingly, we found that the Structure+Flow Index had sufficiently high specificity and sensitivity to discriminate NMOSD–ON eyes from HC eyes. This is consistent with our finding that the retinal microvascular circulation was changed in eyes without a history of ON in NMOSD. According to our results, we speculated that the thinning of pRNFL and GCIPL might be related to abnormalities of the retinal microvascular circulation. These results suggest that primary retinal pathological changes occur in NMOSD. Additionally, the combination of the Macular and Disc Index also had a high diagnostic accuracy of detecting NMOSD eyes that were unaffected by ON. Thus, both the macular and optic disc areas were damaged in NMOSD.

The high diagnostic power of the Structure+Flow Index has scientific and clinical implications. First, the Structure+Flow Index improved our understanding of the pathophysiological mechanisms of NMOSD, suggesting that the presence of retinal vascular changes without previous ON cannot be ignored in patients with NMOSD. Second, vascular parameters might be helpful to explore as an indicator for clinicians to monitor the progression of NMOSD and evaluate the effects of vascular protective drugs in the subclinical phase of this disorder.

There are several limitations in our study. First, we included NMOSD eyes with a contralateral history of ON, although all patients underwent orbital MRI to detect any potential bilateral and chiasmal involvement. Due to our small patient sample size, our findings need confirmation in a larger study. Second, OCT-A is still in rapid development, and the devices and software from different manufacturers are usually not comparable. In the future, OCT-A with better repeatability and reproducibility might provide more stable parameters and image quality to analyze the changes of retinal perfusion in NMOSD.

In conclusion, our current study demonstrated that changes in the retinal microcirculation can occur in NMOSD eyes without a history of ON. The retinal vascular parameters might be helpful to explore as an indicator to discriminate NMOSD eyes without a history of ON. Further clinical trials are warranted to investigate the potential implications of the retinal vascular parameters for the assessment and therapy of this disorder.

## Data Availability Statement

The datasets generated for this study are available on request to the corresponding author.

## Ethics Statement

The studies involving human participants were reviewed and approved by Wenzhou Medical University. Written informed consent to participate in this study was provided by all participants.

## Author Contributions

YC, ZH, LZ, MS, and CS contributed to the study design. YC, CS, SH, and LZ contributed to the data collection. YC, CS, SH, and MS contributed to the data analysis and interpretation. YC, CS, LZ, MS, and ZH contributed to the manuscript preparation. YC, CS, LZ, SH, MS, and ZH contributed to the manuscript revisions. SH, MS, and ZH provided fundings to this research. All authors approved the final version of the manuscript.

### Conflict of Interest

The authors declare that the research was conducted in the absence of any commercial or financial relationships that could be construed as a potential conflict of interest.
