# Corrigendum: The Detection of Retina Microvascular Density in Subclinical Aquaporin-4 Antibody Seropositive Neuromyelitis Optica Spectrum Disorders

**DOI:** 10.3389/fneur.2020.00217

**Published:** 2020-03-31

**Authors:** Yihong Chen, Ce Shi, Lili Zhou, Shenghai Huang, Meixiao Shen, Zhiyong He

**Affiliations:** ^1^Department of Neurology, The Second Affiliated Hospital and Yuying Children's Hospital of Wenzhou Medical University, Wenzhou, China; ^2^School of Ophthalmology and Optometry, Wenzhou Medical University, Wenzhou, China

**Keywords:** neuromyelitis optica spectrum disorders, optical coherence tomography angiography, diagnosis, aquaporin-4 antibody, optic neuritis

In the published article, there was an error in affiliation of author Yihong Chen. As well as having affiliation 1, Yihong Chen should also have affiliation **2**.

The authors apologize for this error and state that this does not change the scientific conclusions of the article in any way. The original article has been updated.

